# De-escalation in breast cancer surgery

**DOI:** 10.1038/s41523-022-00383-4

**Published:** 2022-02-23

**Authors:** Sarah P. Shubeck, Monica Morrow, Lesly A. Dossett

**Affiliations:** 1 Breast Service, Department of Surgery, Memorial Sloan Kettering Cancer Center, New York, NY USA; 2 Division of Surgical Oncology, Department of Surgery, University of Michigan, Ann Arbor, MI USA

**Keywords:** Surgical oncology, Breast cancer

## Abstract

In recent years, several trials of breast cancer treatment have failed to demonstrate a survival benefit for some previously routine surgical therapies in selected patient groups. As each of these therapeutic approaches has been deemed of low value deimplementation has varied significantly. This demonstrates that effective de-escalation in breast cancer surgery relies on more than the availability of data from randomized controlled trials and other high-quality evidence, but is also influenced by various stakeholders, social expectations, and environmental contexts.

Over last 20 years, a growing number of trials of breast cancer treatment demonstrate equivalent survival outcomes when some previously routine therapies, such as axillary dissection and radiotherapy, are omitted in selected patient groups. These findings have led to recommendations for surgical de-escalation by various multidisciplinary oncology groups^1–3^. Current national recommendations for surgical de-escalation in breast cancer care include the avoidance of completion axillary lymph node dissection (ALND) in patients with micrometastases or macrometastases in 1 or 2 sentinel nodes, re-excisions for close but negative surgical margins after partial mastectomy, contralateral prophylactic mastectomy in women with average-risk unilateral breast cancer, and sentinel lymph node biopsy (SLNB) in patients aged >70 years with early-stage hormone receptor–positive (HR + ) cancer (Fig. 1)^2^. Emerging areas of de-escalation include sentinel lymph node biopsy with or without targeted axillary dissection (TAD) after neoadjuvant chemotherapy, and areas under study such as the omission of surgery altogether in selected women with DCIS or in women with clinical complete responses to chemotherapy. As each of these therapeutic approaches can be considered low value (i.e., they incur costs and potentially cause harm while providing no survival benefit), their effective deimplementation remains influenced by various stakeholders, social expectations, and environmental contexts. By examining these deimplementation processes, we can identify factors contributing to the persistent use of low-value services and can leverage this knowledge to design strategies to reduce or eliminate them.

**Fig. 1:**
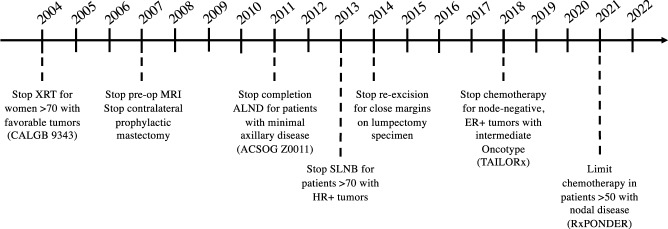
Timeline of national recommendations for breast cancer surgery de-escalation. ALND, axillary lymph node dissection; ER + , estrogen receptor positive; HR + , hormone receptor positive; MRI, magnetic resonance imaging; SLNB, sentinel lymph node biopsy; XRT, radiotherapy.

## Current practices

Surgical procedures incurring costs and possible harms for patients without contributing to improved oncologic or survival outcomes are designated as low value. Harms extend beyond potential short-term surgical complications from the intervention itself to include potential downstream dangerous care cascades (i.e., other tests or treatments that are unlikely to provide value), increased travel burdens for patients and their families, financial toxicity through direct costs and patient-time costs to undergo treatments rather than earn income, and excess health care utilization and spending that may be more appropriately reallocated to high-value services^4,5^.

The management of early-stage breast cancer is ideal for understanding and improving efforts to de-escalate low-value treatments. First, breast cancer is common and well-studied, with many high-quality randomized controlled trials supporting guidelines for de-escalation. Second, broad uptake of screening mammography has resulted in a large cohort of patients with early-stage, highly curable disease who are at risk for overtreatment^6^. Third, breast cancer care is largely decentralized from academic medical centers and is provided in the community, requiring systematic and complete dissemination of de-escalation recommendations. Finally, as breast cancer treatment is inherently multidisciplinary, effective de-escalation of low-value surgery requires the support and coordination of the entire treatment team.

The most broadly disseminated form of value assessment in breast cancer surgery has come from the Choosing Wisely campaign (https://www.choosingwisely.org). Choosing Wisely is an international campaign, initiated by the American Board of Internal Medicine Foundation, that has engaged more than 80 specialty organizations to issue 550-plus recommendations to avoid certain low-value tests or treatments. These guidelines are intended to inform consultations between physicians and patients to facilitate delivery of care that is supported by evidence and is both essential and appropriate. Prominent breast surgical oncology groups, including the Society of Surgical Oncology (SSO), the American Society of Breast Surgeons, and the American College of Surgeons, participate in the campaign and have identified numerous low-value practices. The recommendations specific to low-value breast surgical practices were first issued in 2016 and were updated in 2020; the trials that supported de-escalation and resulted in some practice changes preceded these dates.

There have been several successes in rapid and sustained de-escalation of low-value breast cancer surgery—most notably, the deimplementation of completion ALND for women with minimal nodal disease, after dissemination of the results of the ACOSOG Z0011 trial in 2011^1^. This large randomized controlled trial demonstrated that patients undergoing breast-conserving surgery with metastases in 1 or 2 sentinel lymph nodes did not routinely benefit from completion ALND but could instead be treated with adjuvant radiotherapy. After dissemination of the trial results at national meetings in 2010 and in a landmark publication in 2011, ALND was substantially de-escalated in appropriate populations within 18 months. Specifically, the rates of ALND use in patients with minimal nodal disease decreased from 63% in 2004 to 14% in 2016, with the most substantial decrease occurring between 2010 and 2011, corresponding to the release of the Z0011 results^7^. This decrease in the rate of ALND use was noted across care environments and in several countries^8–10^. Factors facilitating this rapid and sustained deimplementation include the broad dissemination of study findings, the opportunity to avoid operative morbidity and chronic lymphedema, and provider confidence in the strength and quality of the recommendation^8^.

A similar success occurred in 2013, when the SSO and the American Society for Radiation Oncology released an evidence-based consensus statement defining an adequate surgical margin in breast-conserving surgery^2^. The consensus panel determined that margins more widely clear than “no ink on tumor” did not reduce rates of local recurrence and were not routinely necessary. This recommendation allowed for avoidance of reoperation in women with a close but negative margin after partial mastectomy, a practice accounting for approximately half of the re-excisions performed at the time^11^. In the 18 months after the dissemination of this guideline, there was a substantial and rapid decrease in reoperations, both re-excisions and subsequent conversions to mastectomy^12–14^. This decrease has persisted over time and has been seen in both population-based and single-institution studies. Several factors led to wide acceptance of this recommendation, including the evidence supporting the recommendation, the decreasing rates of locoregional recurrence observed over time that were attributable to differences in systemic therapy and tumor biology, the ability to avoid returning a patient to the operating room, and the clear definition of what constitutes an adequate margin, which facilitated patient discussion and pathology review^8^.

In contrast to these successful deimplementation efforts, some breast surgical practices designated as low value remain common despite national recommendations supporting their omission. These include contralateral prophylactic mastectomy (CPM) in women with average-risk unilateral breast cancer and SLNB in women aged >70 years with HR + , HER2-negative early-stage breast cancer^7^. Similar to ALND for minimal nodal disease and re-excision for close but negative surgical margins, these practices expose patients to harms and costs without providing a survival benefit, which has resulted in national recommendation to avoid their use. The SSO first issued a consensus guideline in 2007 recommending the avoidance of CPM in patients with average-risk unilateral cancer^15^. Although CPM does reduce the risk of developing a contralateral breast cancer, it affords no additional survival benefit, compared with unilateral breast surgery, in patients with average-risk disease. Despite the 2007 recommendation to avoid this practice, rates of CPM for patients with average-risk disease have continued to steadily increase, from 11% in 2004 to 26% in 2016^7,16^. When surgeons describe their justifications for the continued use of this approach, they often emphasize patient autonomy, perception of improved patient psychological well-being after surgery, and patient preference^8,17^. Patients who opt for bilateral mastectomy similarly cite concerns about the development of a future breast cancer and associated anxiety. However, psychosocial outcomes are not better after CPM than after unilateral breast surgery^18,19^. Even with this lack of survival and psychological benefits for patients, use of CPM continues to increase, in contrast to recommended practice.

A similar pattern has been observed for SLNB in older women with early-stage HR + cancer. Among patients in CALGB 9343, which randomized women aged >70 years with HR + clinical stage I breast cancer to tamoxifen plus radiation therapy or tamoxifen alone after lumpectomy, radiation therapy conferred a small benefit in the rate of locoregional recurrence but did not result in longer overall survival. Furthermore, there was no difference in survival was identified in patients who did and did not undergo axillary evaluation at the time of surgical excision. In long term follow up of the CALGB 9343 cohort, a 3% increase in axillary recurrence was noted in those who did not undergo radiation therapy or SLNB. Importantly, the result of SLNB in this cohort is unlikely to impact systemic therapy choice^7,20^. Despite publication of these findings in 2013 and the subsequent recommendation in 2016 to avoid SLNB in patients aged >70 years with HR + breast cancer, the rate of use of this low-value procedure has remained relatively stable: 88% in 2013 and 87% in 2016^7^. Qualitative evaluation of patients and providers revealed skepticism regarding supporting data, uncertainty about broad application of age-specific cutoffs, and perceptions that SLNB results in minimal morbidity as justifications for the continued use of SLNB. For example, patients and providers often dispute set age cutoffs in favor of assessment of an individual patient’s physiology. Finally, similar to the reasons underlying the continued use of CPM, the continued use of SLNB has been justified in terms of promoting patient peace of mind and allowing the determination of nodal involvement to confirm cancer staging^8,21^.

In addition to the guideline alterations and shifts in practice described above, there are other emerging areas of surgical de-escalation that have impacted the surgical care of patients with breast cancer. For example, in node positive patients undergoing neoadjuvant chemotherapy, sentinel lymph node biopsy had been demonstrated to be a reliable method of assessment of axillary disease burden when dual tracer was utilized and three or more sentinel nodes are identified, with a false negative rate of <10%^22^. This has allowed for omission of ALND in node positive patients who downstage after neoadjuvant chemotherapy. Additionally, in node positive patients undergoing neoadjuvant chemotherapy, the clipping of positive nodes to allow for their removal at the time of surgery when they are not sentinel nodes has been demonstrated to further reduce the false negative rate in retrospective studies^23^. These approaches allow avoidance of ALND in 50–80% of node positive patients with triple negative and HER2 positive cancers^24^.

### Deimplementation strategies

The differing outcomes among deimplementation efforts across four low-value breast surgical practices is a clear demonstration that effective de-escalation in breast cancer surgery relies on more than the availability of data from randomized controlled trials and other high-quality evidence. With substantial differences in the contexts of breast cancer care delivery and the strong emphasis on patient autonomy in decision-making, breast cancer surgery practices remain influenced by social, economic, and other factors that may affect deimplementation initiatives^25^. To uniformly and sustainably reduce the use of low-value practices, avoid overtreatment, and deliver the highest-quality evidence-driven care, deimplementation in breast cancer surgery will require strategies specifically targeted at the patient, physician, and society levels. These strategies may differ from those commonly used to reduce the use of low-value diagnostic testing or medications, such as ordering or formulary restrictions or market withdrawal.

Deimplementation in breast cancer surgery should account for patients’ fears and, often, misperceptions of the risks of cancer recurrence, which can lead to patients being inclined to undergo more-invasive surgical interventions. For example, patients and their advocates may have concerns other than overall survival, such as fear of local recurrence or the desire to have peace of mind with their surgical decision. In these scenarios, patients may opt for treatments that do not necessarily convey a survival advantage but rather lead to reduced rates of local recurrence or contralateral breast cancer^21,26^. Additionally, even when patients report being aware of a lack of survival advantage in opting for more invasive surgery, patients still cite desire to extend their life as key to their decision making^27^.

Initial surgical treatment decisions may not fully consider the long-term harms or be able to anticipate potential decisional regret of choosing more invasive surgical treatment for breast cancer. For example, in consideration of contralateral prophylactic mastectomy in patients with nonhereditary breast cancer, patients often aim to reduce anxiety of recurrence through more invasive surgery, but may not actually benefit from reduced recurrence fear and may experience worse body image and quality of life following surgery^28,29^. Patients considering more invasive surgical options may be best served by the use of patient education tools that emphasize communication of risks and introduce literature reporting on long term quality of life when comparing surgical interventions with comparable oncologic outcome. These efforts may help patients balance their desire for an overall excellent prognosis and low risk of recurrence with the potential costs and harms of overtreatment.

Clinicians often overestimate the benefits of procedures, underestimate the potential harms or costs of unnecessary intervention, and do not consider or misinterpret their patients’ values or desires^8,21^. Such clinicians may benefit from exposure to data, beyond the results of randomized controlled trials, focusing on the interaction of patient satisfaction and psychological outcomes after surgery. In addition to educational interventions, clinician-level strategies (including peer comparison, audit and feedback, and provider-directed financial incentives) may play a role in future deimplementation efforts in breast cancer surgery. On a broader scale, payment reform or a transition to value-based insurance may further influence decision-making in breast cancer surgery and promote the deimplementation of low-value practices. For example, payers may opt to incentivize facilities with high levels of appropriateness per current recommendations or implement value-based payment designs where low-value treatments may incur greater out of pocket costs to patients. Additionally, accreditation measures may have greater emphasis on appropriateness of care with collecting and reporting of data related to low-value practices.

Importantly, de-escalation of breast cancer surgery must be conducted and monitored in the context of multidisciplinary care. As surgical practices are de-implemented, there may be a tendency toward escalation of adjuvant therapies. Specifically, in cases of uncertain nodal involvement when criteria for omission of SLNB was met, radiation may be more frequently offered or genomic assays more often used. These challenges in coordination between multidisciplinary care team members will be inherent in the changing landscape of breast cancer care and collaboration essential in order to resist unnecessary and potentially harmful care.

### Future directions in de-escalation of breast cancer surgery

As the trend for less-intensive surgical interventions for breast cancer continues, the need for deimplementation will persist. Investigators are currently evaluating the safety of omitting surgery in the context of a complete clinical response to neoadjuvant therapy for invasive cancer, as well as the safety of omitting surgery from the management of patients with ductal carcinoma in situ^30–32^. However, the criteria for omission of surgery will likely spare only a minority of patients from undergoing excision, and the additional cost in terms of biopsies and surveillance and potentially increased anxiety may counteract any potential benefits of surgical de-escalation^33^. The presence of nonoperative trials signals that breast surgical oncologists will continue to be challenged to respond to the growing body of data, which may result in recommendations for even less surgery for patients with breast cancer.

Beyond interpreting the evidence from trials, the ability to successfully deimplement low-value breast cancer surgery will require an ability to adapt to new evidence, understand what is meaningful value for patients, and consider the interests and needs of all stakeholders^34^.

### Reporting summary

Further information on research design is available in the Nature Research Reporting Summary linked to this article.

## Supplementary information

Reporting Summary Checklist
